# The Effect of CM082, an Oral Tyrosine Kinase Inhibitor, on Experimental Choroidal Neovascularization in Rats

**DOI:** 10.1155/2017/6145651

**Published:** 2017-10-22

**Authors:** Chengda Ren, Hui Shi, Juanjuan Jiang, Qingyu Liu, Yaru Du, Mengmei He, Wenting Cai, Qingquan Wei, Jing Yu

**Affiliations:** ^1^Department of Ophthalmology, Shanghai Tenth People's Hospital, Tongji University School of Medicine, Shanghai 200072, China; ^2^Department of Cardiac Surgery, Institute of Cardiovascular Diseases of Fudan University, Affiliated Zhongshan Hospital of Fudan University, Shanghai, China; ^3^DMPK & Bioanalysis Department Sundia MediTech Company, Shanghai, China; ^4^Department of Ophthalmology, Shanghai Tenth People's Hospital, Shanghai, China; ^5^Department of First Clinical Medical College, Nanjing Medical University, Nanjing, China; ^6^Department of Ophthalmology, Shanghai Tenth People's Hospital, Nanchang University, Nanchang, China

## Abstract

The aims of this study were to evaluate the effects of CM082 on the development of choroidal neovascularization (CNV) in a laser-induced CNV rat model and to determine the drug concentration in the ocular tissues. After the laser-induced CNV model was established in rats, CM082 was orally administered. The effects of CM082 on the CNV lesions were assessed using fundus fluorescein angiography (FFA), CNV histology, and retinal pigment epithelium- (RPE-) choroid-sclera eyecup analysis. The concentrations of CM082 in the plasma and eye tissues were determined using liquid chromatography-tandem mass spectrometry (LC-MS/MS). Results of FFA, histology, and RPE-choroid-sclera eyecup analysis demonstrated that the CM082-treated (10 mg/kg/d or 30 mg/kg/d) rats exhibited significantly less neovascularization than did the control group. The total concentration of CM082 in the eyes (172.86 ± 57.11 ng/g) was similar to that in the plasma (196.87 ± 73.13 ng/ml). Within the eye, the concentrations of CM082 and its metabolites were highest in the retina-sclera. The orally administered CM082 thus effectively passed through the blood-retina barrier (BRB) to reach the retina in the Brown Norway rats. Therefore, at both 10 mg/kg/d and 30 mg/kg/d, CM082 was able to reduce CNV lesions in the laser-induced CNV rat model.

## 1. Introduction

Age-related macular degeneration (AMD) is one of the most common causes of irreversible central visual loss in people over 65 years of age in Europe and North America [1, 2]. There are two different subgroups of AMD: the geographic atrophy, or dry, form and the chronically neovascularized, wet form. Although the wet form accounts for only 10% of AMD cases, it is the main cause of visual loss in 60–80% of AMD patients [3, 4]. Therefore, it is necessary to understand the pathology of choroidal neovascularization (CNV) and to identify efficient therapies.

A large number of studies have indicated that neovascularization is caused by the overexpression of vascular endothelial growth factor (VEGF), a growth factor that plays a major role both in the development of normal blood vessels and in abnormal angiogenesis [5–7]. There are five VEGF isoforms (VEGF-A, B, C, D, and E) as well as placental growth factor (PIGF) in the VEGF family [8, 9]. VEGF has to bind to one or more VEGF receptors (VEGFRs) to exert its function [10]. When bound to one or more VEGFs, VEGFR autophosphorylates and dimerizes to phosphorylate the specific intracellular tyrosine residue that activates the signaling pathway, which then specifically promotes the mitosis and proliferation of vascular endothelial cells and regulates their migration and survival [11, 12]. Anti-VEGF therapies that reduce the interaction of VEGF with its receptors, such as ranibizumab [13], aflibercept [14], and bevacizumab [15], are widely used to treat patients with CNV secondary to AMD and other pathological conditions. Reducing VEGF-A binding to VEGFRs, and especially VEGFR-2, is the main target of ranibizumab and bevacizumab [16]. Although these therapeutic drugs are relatively effective for treating AMD and related eye diseases, not all patients respond to them and many exhibit decreased drug susceptibility during treatment [17]. Additionally, repeated intravitreal injection can cause rare but serious side effects such as ocular pain, infection, or hemorrhage. To avoid the intravitreal injection-related complications and relapse, it has been necessary to develop a less invasive treatment.

In addition to VEGF, a growing body of evidence indicates that platelet-derived growth factor (PDGF) contributes to neovascularization in AMD [18]. PDGF plays a role in angiogenesis by recruiting pericytes to the newly formed blood vessels and maintaining the stabilization and maturation of blood vessels. Furthermore, pericyte-derived VEGF and cell-cell contacts may participate in promoting endothelial survival and may guide migration. The previously established endothelial/pericyte associations and vessel stabilization are disrupted when PDGF/PDGF receptor (PDGFR) signaling is inhibited [19]. Considering the synergistic effects of VEGF and PDGF signaling, therapeutic methods of inhibiting both the VEGF and the PDGF pathways using two biologics (e.g., ranibizumab and Fovista) are being actively investigated [20, 21]. Nevertheless, phase 3 clinical trial demonstrated that the addition of Fovista to a monthly Lucentis regimen did not result in benefit as measured by the mean change in visual acuity at the 12 month time point. It is necessary to develop a better understanding in anti-PDGF therapies for AMD.

CM082 is a multitarget tyrosine kinase inhibitor that can suppress neovascularization by inhibiting the VEGF, PDGF, c-kit, and Flt-3 receptor tyrosine kinases. CM082 is a novel derivative of sunitinib that has been approved for the treatment of cancers and that was designed to have a more favorable toxicity profile than sunitinib. The oral administration of CM082 is more convenient than intravitreal injection, and its inhibition of both VEGFR and PDGFR might be more effective than anti-VEGF injections alone. Tyrogenex has completed a phase 1 clinical study of X-82 (CM082) in the USA and is currently conducting a randomized phase 2b study in patients with exudative AMD [22] (NCT02348359). Meanwhile, AnewPharma is conducting a phase 1 study in China (NCT02452385). Here, we report the effect of orally administered CM082 on CNV lesions in a rat model and describe the concentration of CM082 in the ocular tissues.

## 2. Materials and Methods

### 2.1. Compound

CM082 (lot 20100111-B) was provided by Tyrogenex, Inc. (Palm Beach Gardens, FL, USA). It was formulated as a suspension in 0.5% HPMC-K4 M and 0.2% SLS in double-distilled water. The concentration was 2 or 6 mg/ml, and it was stored at 4°C. A dose of 10 mg/kg/d or 30 mg/kg/d was administered by oral gavage, with a dosing volume of 5 ml/kg.

### 2.2. Animals

A total of 70 male Brown Norway (BN) rats (age, 10 weeks; weight, 200 ± 20 g) were used. All rats were handled in compliance with the ARRIVE guidelines. All animal experiments were approved by the Institutional Animal Care and Use Committee of the College of Medicine, Tongji University, Shanghai, China.

### 2.3. Laser-Induced CNV in Rats

Laser photocoagulation-induced CNV was established as previously described [23]. Preoperative preparation included general anesthesia, which was induced with an intraperitoneal injection of 1% pentobarbital sodium (40 mg/kg; Sigma-Aldrich, St. Louis, MO, USA). The rats' pupils were dilated using 0.5% tropicamide and 0.5% phenylephrine (Mydrin-P, Santen Pharmaceutical Co., Osaka, Japan). Surface anesthesia was induced with 0.5% Alcaine (Alcon (China) Ophthalmic Product Co., Beijing, China). Bruch's membrane of the right eye was injured using the following laser parameters: 532 nm wavelength, 360 mW intensity, 0.1 s duration, and 50 *μ*m spot size. Eight to ten laser spots were applied to the major retinal vessels at approximately the same distance to the optic disc. A laser-induced cavitation bubble or slight hemorrhage indicated a rupture in Bruch's membrane. Fundus photography was taken immediately after laser photocoagulation to check for fundus hemorrhage.

### 2.4. CM082 Treatment of Experimental CNV

To assess the CM082 distribution and the inhibitory effects of CM082 on CNV development, the 70 rats were randomly divided into 2 groups: 10 rats for evaluating the CNV rat model and to detect the distribution of CM082 and its metabolites and 60 rats for assessing the effects of CM082 on the experimental CNV. CM082 was administered orally at dose of 10 mg/kg/d or 30 mg/kg/d, while the vehicle (5 mg/kg/d) was used as a negative control.

### 2.5. LC-MS/MC Analysis

Liquid chromatography-tandem mass spectrometry (LC-MS/MS) was employed to detect the concentrations of CM082 and its metabolites (X-297 (C_22_H_24_FN_5_O_3_) and X-471 (C_23_H_28_FN_5_O_4_)) in the plasma and ocular tissues. An API-4000 triple-quadrupole mass spectrometer (AB Sciex, Framingham, MA, USA) coupled with a Shimadzu liquid chromatography system (Shimadzu Cooperation, Kyoto, Japan) was used for the analysis. CM082, its metabolites, and tolbutamide were dissolved in DMSO to generate 1.0 mg/ml stock solutions stored at 4°C. A total of 200 *μ*l of the tolbutamide stock solution was dissolved in acetonitrile to produce internal-standard working solutions. The plasma and ocular tissue samples were analyzed along with the standard and quality-control samples. The data were analyzed with Analyst 1.6.1 (AB Sciex, Framingham, MA, USA).

### 2.6. Fluorescence Angiography

Fundus fluorescence angiography (FFA) was performed on days 7, 14, and 21 after laser photocoagulation. The preoperative preparations were the same as those used to establish the CNV model. Approximately 0.2 ml of 10% sodium fluorescein (Alcon Japan, Tokyo, Japan) was injected intraperitoneally, and the leakage of fluorescein from the laser lesions was monitored dynamically for up to 15 min. The images were obtained using confocal scanning laser ophthalmoscopy (Spectralis, Heidelberg Engineering Inc., Heidelberg, Germany) and analyzed using ImageJ software (National Institutes of Health, Bethesda, MD, USA). The signal intensities (brightness of the CNV lesions) defined the degree of leakage. As a reference, the intensity of the capillaries in the normal regions was defined as 0, whereas the intensity at the major branch of the retinal vein was defined as 1.

### 2.7. CNV Histology and Immunohistochemistry

The rats were euthanized after the FFA examination on day 14 or 21 after laser injury, and a histological examination was performed on 4 rats per group at each time point. The animals were enucleated, and the eyes were fixed in paraformaldehyde for 24 h (the eyelashes were also removed). After fixation, the eyes were embedded in paraffin and serially sectioned at 5 *μ*m. Routine hematoxylin-eosin (HE) staining was performed on selected slides, and the serial sections were examined using a light microscope. Immunohistochemical staining was performed on the histological sections from the CM082-treated and vehicle-treated groups 14 and 21 days after laser administration. Routine immunolocalization procedures were used to dewax and rehydrate the slides. The slides were incubated with 3% hydrogen peroxidase for 25 min and washed in phosphate-buffered saline (PBS) for 5 min. The slides were then blocked in goat serum for 15 min. Next, the sections were incubated with a phosphorylated anti-VEGFR-2 antibody (Nanjing Bioworld Biotech Co., Jiangsu, China) overnight at 4°C, followed by incubation with Goat Anti-Rabbit/Rat IgG antibody (Dako Denmark A/S, Denmark) for 30 min. The sections were subsequently incubated with horseradish peroxidase-conjugated streptavidin (Thermo Fisher Scientific China, Shanghai, China) for 30 min and then counterstained with hematoxylin for 2 min. Finally, the slides were mounted in aqueous mounting medium and examined using ScanScope.

### 2.8. Measurement of the CNV Area

To assess the inhibitory effects of CM082 on CNV development, the photocoagulated rats were randomly divided into 2 groups: (1) a CM082-treated group (*n* = 9) and (2) a vehicle-treated group (*n* = 9). CM082 (30 mg/kg/d or 5 ml/kg/d) or vehicle (5 ml/kg/d) was administered beginning on the 7th day after laser injury. On the 7th, 14th, or 21st day, 3 rats from each group were anesthetized, and the left ventricle of the heart was perfused with 2 ml of PBS containing 50 mg of fluorescein isothiocyanate- (FITC-) dextran (2 × 10^6^ average molecular weight; Sigma-Aldrich). The enucleated eyes were fixed in 4% paraformaldehyde for 1 h. Retinal pigment epithelium- (RPE-) choroid-sclera flat mounts were then produced by hemisecting the eye, and the neural retina was peeled away from the underlying RPE. Radial cuts were performed to permit the tissue to be flattened onto a microscope slide, with the RPE side facing up, after which the CNV lesions in the flat mounts were examined by scanning laser confocal microscopy (Zeiss, Jena, Germany). The CNV area was measured by ImageJ software (National Institutes of Health, Bethesda, MD, USA).

### 2.9. Statistical Analysis

All statistical graphs were generated in GraphPad Prism 5.0 (GraphPad Software, Inc., San Diego, CA, USA). The intensity of leakage detected by FFA and the CNV lesion areas detected following the FITC-dextran perfusion were assessed with ImageJ (National Institutes of Health, Bethesda, MD, USA) and evaluated with one-way analysis of variance (ANOVA) and Scheffe's multiple comparison tests using SPSS (SPSS version 20.0, Chicago, IL, USA). The results are presented as the mean ± SEM, unless otherwise stated, and box plots are used to graphically display the data from the different groups. In this study, *p* ≤ 0.05 was considered statistically significant.

## 3. Results

Sixty-eight BN rats were used in this study. Two of them were discarded because of large areas of fundus hemorrhage after laser injury, and another one had a slight subretinal hemorrhage, although the hemorrhage was absorbed at 7 days after the laser injury. None of the rats in our research exhibited conjunctival hemorrhage, corneal opacity, cataracts, retinal detachment, or an anesthesia accident. All rats tolerated the CM082 treatment well, and there was no behavioral change, death, or body weight loss during treatment.

### 3.1. CNV Rat Model Establishment and Distribution of CM082 in the Eye

**Figure 1:**
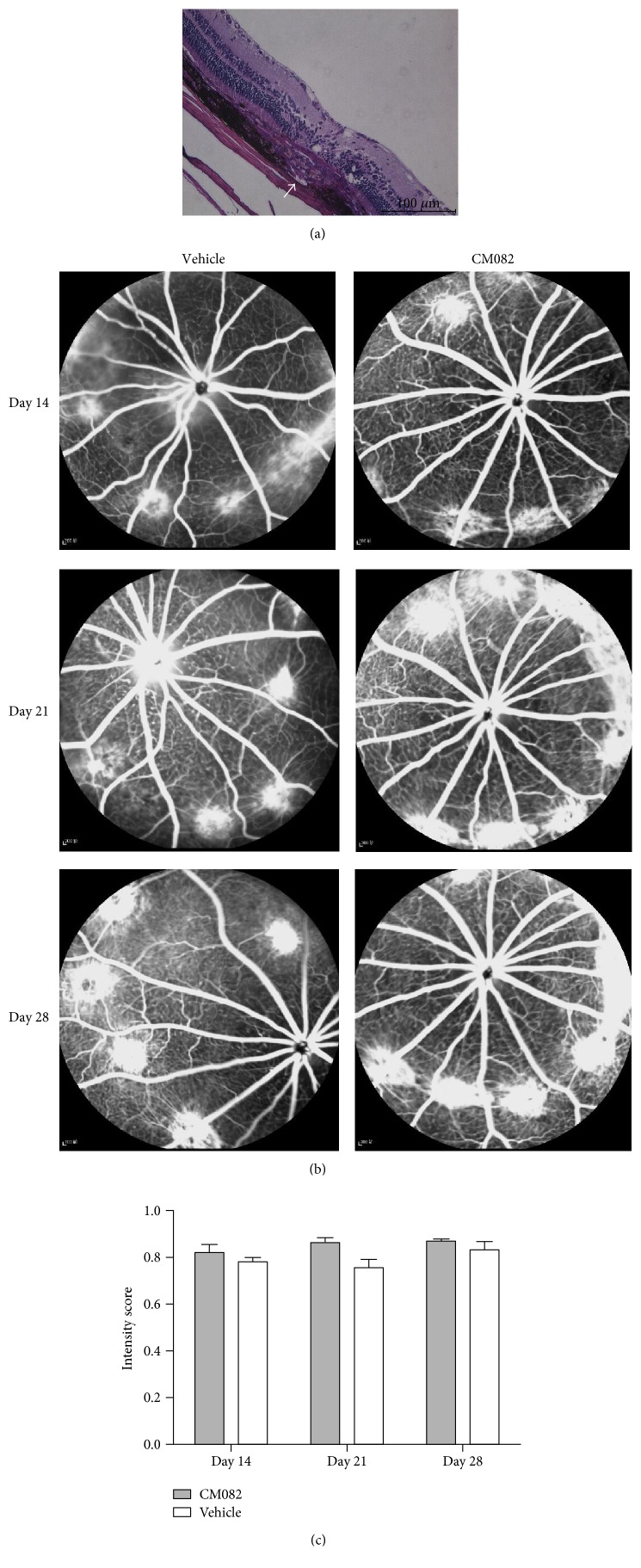
Histology and FFA results of CNV modeling. (a) Histology of CNV lesions stained with HE shows that CNV formation and broke the Brunch's membrane at 21 days after photocoagulation. Dome-like CNV complexes, consisting of fibrovascular tissue, retinal pigment epithelial cells, and pigment clumps, are shown and white arrow indicates the vessel lumina of neovascular. (b) FFA results showed fluorescein leakage of CNV in each group at day 14, 21, and 28 (7 days after CM082 treatment) separately. The signal intensity of capillary in background region was defined as “0” whereas the signal intensity of the main branch of the retinal vein was defined as “1”. (c) There is no significant difference between the intensity score of leakage in each group at day 14, 21, and 28 (*p* > 0.05). Scale bar, 100 *μ*m.

**Figure 2:**
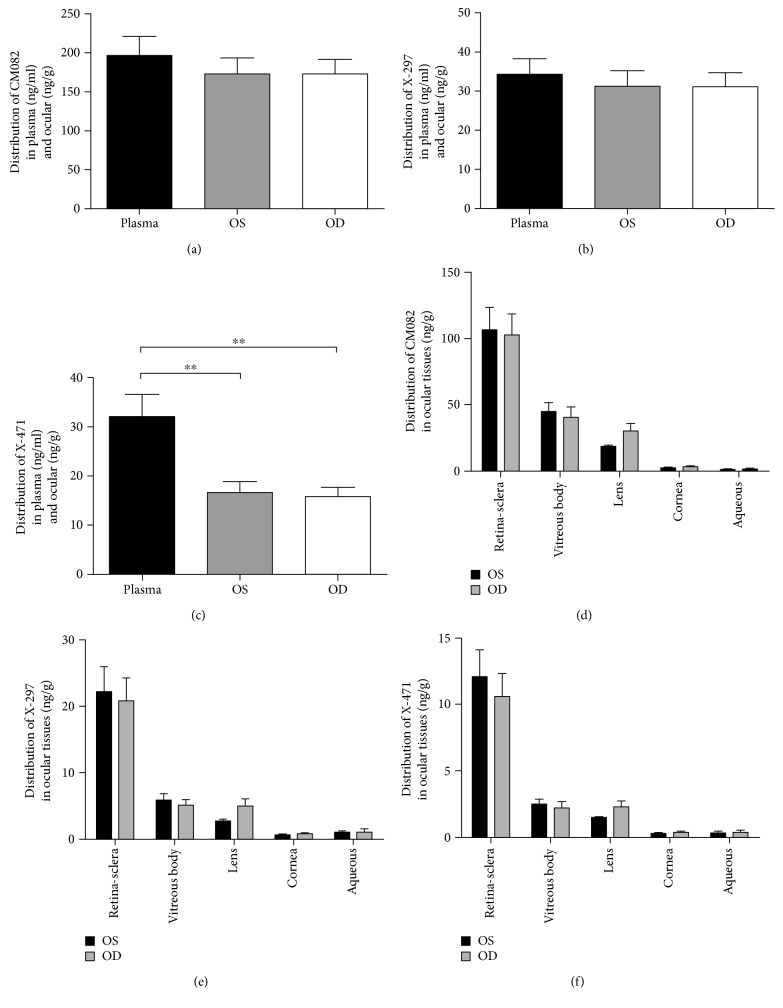
Distribution of CM082 and its metabolites in plasma and eye tissues. (a, b, and c) The concentrations (ng/ml or ng/g) of CM082, X-297, and X-471 in plasma and ocular tissues (*n* = 9). There is no significant difference between the concentration of CM082 in plasma and eyes. The same is true for X-297. However, the concentration of X471 in eyes (OS: 16.68 ± 2.18, OD: 15.82 ± 1.85) is obviously lower than that in plasma (32.04 ± 4.57) (^∗∗^*p* < 0.01). (d, e, and f) The concentration of CM082, X-297, and X-471 was detected in different ocular tissues (*n* = 9). There is no significant difference in concentration of CM082, X-297, and X-471 between OS and OD.

Ten BN rats were photocoagulated in the oculus dexter (OD) to build the CNV rat model. Histological examination was performed at day 21, while FFA was conducted at days 14 and 21 to ensure that the CNV model was established successfully and to provide evidence that neovascularization was induced (Figures 1(a), 1(b), and 1(c)). After modeling CNV successfully, to investigate whether CM082 and its metabolites (X-297 and X-471) can reach the retina effectively, the rest of the nine rats were orally treated with CM082 at 10 mg/kg/d for 9 days, starting from day 22. FFA was performed after 7 days of CM082 administration, but there was no significant reduction in fluorescein leakage compared to leakage at days 14 and 21 (Figures 1(d) and 1(e), *p* > 0.05). This result may have been due to the fact that CM082 was administered at day 22, when the CNV had formed completely and irreversibly. Two days after FFA, the rats were sacrificed, and we determined the concentrations of CM082 in the plasma (ng/ml) and the ocular tissues (ng/g) 2 h after CM082 treatment. The concentrations of CM082 in the plasma, the oculus sinister (OS), and the OD were 196.87 ± 24.38 ng/ml, 172.74 ± 20.83 ng/g, and 172.97 ± 18.33 ng/g, respectively. The corresponding concentrations of X-297 were 34.42 ± 3.86 ng/ml, 31.28 ± 4.00 ng/g, and 31.11 ± 3.56 ng/g, while those of X-471 were 32.04 ± 4.57 ng/ml, 16.68 ± 2.18 ng/g, and 15.82 ± 1.85 ng/g. Although the concentrations of CM082 and X-297 in the plasma were slightly higher than those in the ocular tissues, the difference was not significant, demonstrating that CM082 and X-297 can both enter the ocular tissues effectively (Figures 2(a) and 2(b), *p* > 0.05). However, the concentration of X-471 in the plasma was significantly higher than that in the ocular tissues (Figure 2(c)), suggesting that X-471 may be less effective at passing through the blood-retina barrier (BRB). Within the ocular tissues, the distributions of CM082, X-297, and X-471 were highest in the retina-sclera 2 h after CM082 administration. There was no significant difference in the drug concentrations between the OS and the OD (Figures 2(d), 2(e), and 2(f)), indicating that the laser injury did not affect the drug distribution in the eyes. These results showed that CM082, X-297, and X-471 (to a lesser extent) can successfully pass through the BRB and reach the retina.

### 3.2. Regression of Established CNV after CM082 Application

To determine whether CM082 inhibited CNV progression, 60 BN rats were divided into 4 groups and randomized by weight, with 10 rats receiving CM082 at 10 mg/kg/d, 10 receiving CM082 at 30 mg/kg/d, 20 receiving vehicle treatment, and 20 undergoing the RPE-choroid-sclera preparation. Either CM082 or vehicle was administered beginning on the 7th day after laser injury. The CNV analysis was performed on day 14 or 21 after laser photocoagulation using FFA, histological examinations, and immunohistochemistry.

#### 3.2.1. Effects of CM082 on CNV at a Dose of 10 mg/kg/d

**Figure 3:**
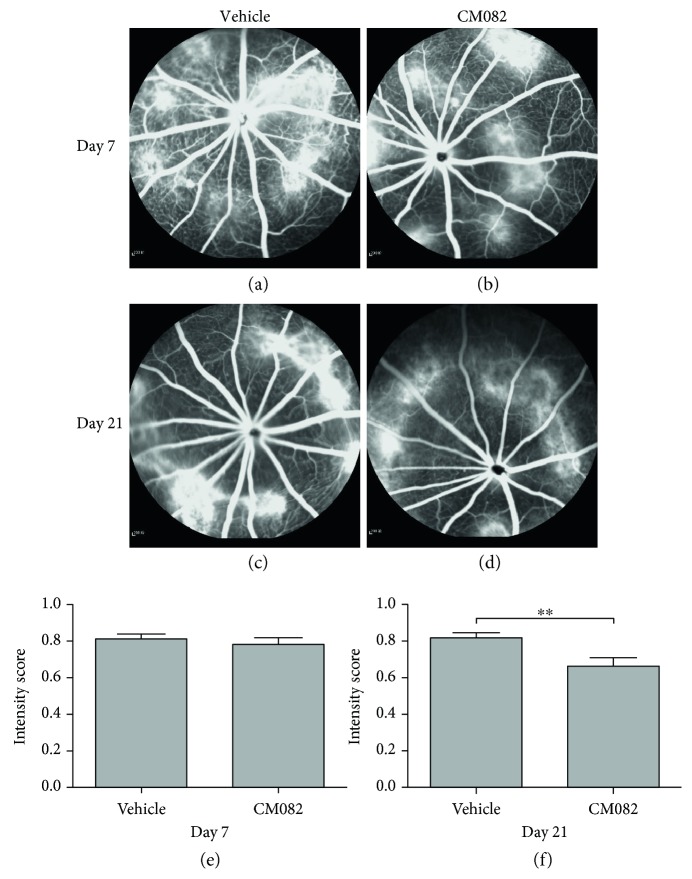
FFA results of the CM082 (10 mg/kg/d) group and the corresponding vehicle-treated group at different time points. (a, b; *n* = 10) FFA showed that the leakage of fluorescein at laser spots are at nearly same extent in vehicle-treated and CM082 group 7 days after laser injury (*p* > 0.05, *n* = 9). (c, d) Leakage of fluorescein in the CM082-treated group was reduced at 21 days after laser injury (^∗∗^*p* < 0.01, *n* = 9).

The group receiving 10 mg/kg/d CM082 was analyzed by FFA before CM082 administration. The results on days 7 and 21 after laser injury are shown in Figure 3. The FFA results on day 7 were similar between the ultimately vehicle-treated and CM082-treated groups (vehicle: 0.81 ± 0.03, CM082: 0.78 ± 0.04; Figures 3(a), 3(b), and 3(e)). We then began to treat the rats with CM082 at 10 mg/kg/d, and FFA was again performed at day 21 (14 days after CM082 dosing). The leakage in the CM082-treated group was significantly lower than that in the vehicle-treated group (vehicle: 0.82 ± 0.03, CM082: 0.66 ± 0.05; Figures 3(c), 3(d), and 3(f)), which indicated that oral administration of CM082 at 10 mg/kg/d can reduce CNV leakage.

**Figure 4:**
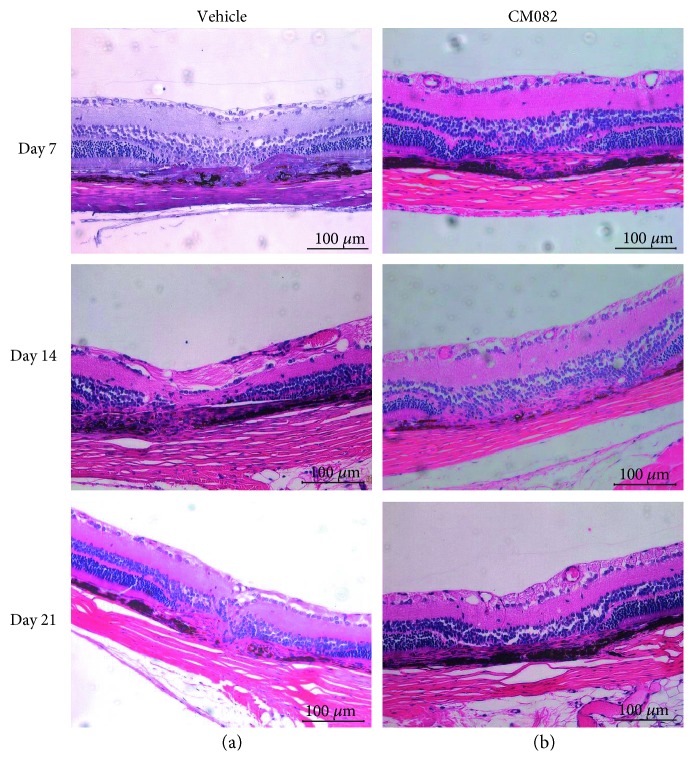
Histology examine stained with HE of the CM082 (10 mg/kg/d) group (*n* = 2) and the corresponding vehicle group (*n* = 2) was obtained after FFA examination at day 7, 14, and 21. (a, b) Results showed a smaller CNV size of the CM082-treated group at day14 and 21. CNV complexes consist of retinal pigment epithelial cells, pigment clumps, and vascular tufts were observed. The CNV under CM082 treated was thinner in the center compared with that in the vehicle-treated group. Scale bar, 100 *μ*m.

We further demonstrated the effects of CM082 using histological examination of the retina of BN rats. At day 21, the CNV lesions were smaller, and there was less CNV complex (Figure 4). The results showed not only an inhibitory but also a regressive effect of CM082 on CNV development and suggested that orally treating BN rats with CM082 at 10 mg/kg/d can reverse CNV without significant toxicity.

#### 3.2.2. Effects of CM082 on CNV at a Dose of 30 mg/kg/d

**Figure 5:**
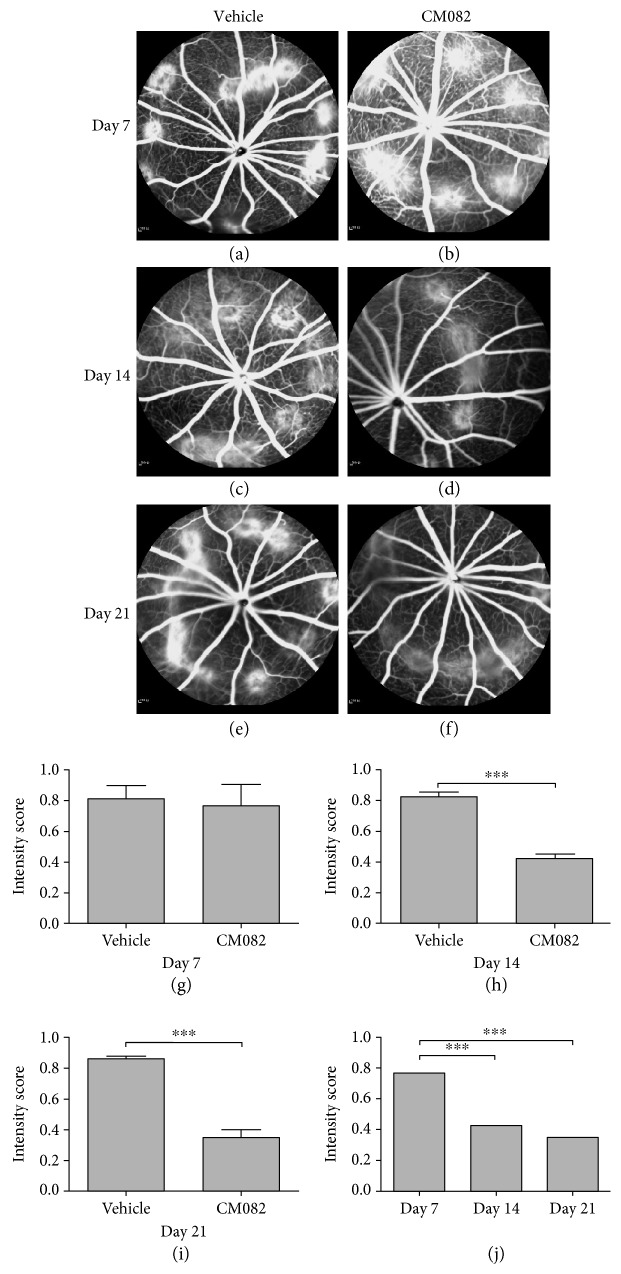
FFA results of the CM082 (30 mg/kg/d)-treated group and the corresponding dose of the vehicle-treated group at different time points. Results of FFA showed the leakage of fluorescein at laser spots in vehicle-treated and CM082 group 7 (a, b; *n* = 10), 14 (c, d; *n* = 9), and 21 (e, f; *n* = 8) days after laser injury. (g, h, and i) The fluorescence signal intensity of CM082-treated group was statistically lower than the vehicle-treated group at 14 days and 21 days (^∗∗∗^*p* < 0.001). (j) The signal intensity at 14 and 21 days was obviously lower than that at 7 days in the CM082 group (^∗∗∗^*p* < 0.001).

To investigate whether CM082 can be administered at a higher dose, we treated BN rats with CM082 at 30 mg/kg/d 7 days after photocoagulation. Before dosing, we performed FFA to ensure that CNV leakage was occurring in the two groups at the approximate baseline (vehicle: 0.81 ± 0.02, CM082: 0.77 ± 0.04; Figures 5(a), 5(b), and 5(g); *p* > 0.05). After CM082 administration, the results on days 14 and 21 showed that the fluorescein leakage in the CM082-treated group (day 14: 0.42 ± 0.03, day 21: 0.35 ± 0.05) was significantly reduced compared to that in the vehicle-treated group (day 14: 0.83 ± 0.03, day 21: 0.86 ± 0.02; Figures 5(c), 5(d), 5(e), 5(f), 5(h), and 5(i); ^∗∗∗^*p* < 0.001). More importantly, while the intensities in the vehicle-treated group increased slightly over time (indicating disease progression), the intensities in the CM082-treated group decreased over time, suggesting that CM082 can not only inhibit CNV progression but also cause regression of CNV lesions (Figure 5(j)).

**Figure 6:**
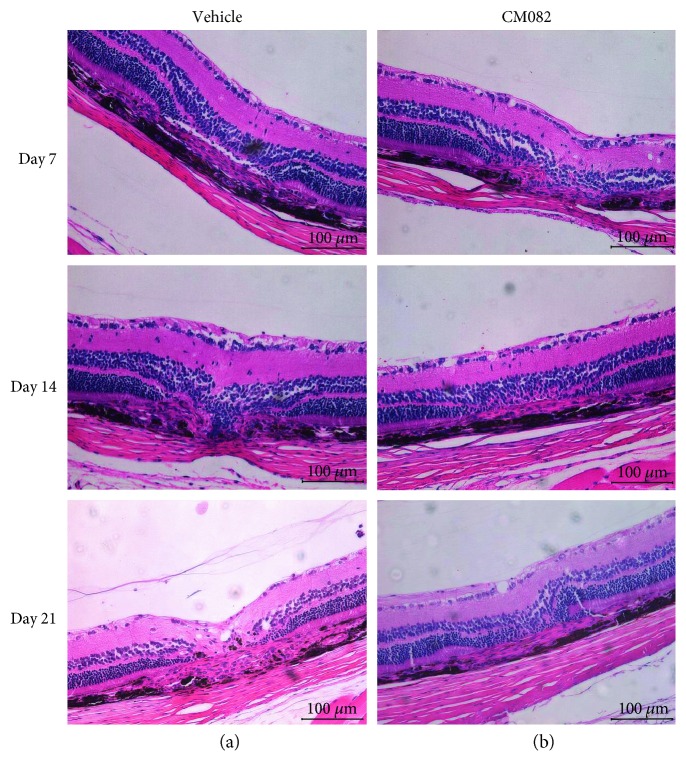
Histology of CNV lesions stained with hematoxylin-eosin of the CM082 (30 mg/kg) group (*n* = 6) and the corresponding dose of the vehicle group (*n* = 6) was obtained after FFA examination. Results showed that administration of CM082 reduces laser-induced CNV lesions. (a) In the vehicle-treated group, CNV formed and broke the Brunch's membrane. The depigmentation in RPE, aggregation of macrophage, and neovascularization were obtained 14 days and 21 days after photocoagulation. (b) In the CM082 group, the edema, depigmentation, and CNV areas significantly decreased comparing to vehicle-treated group at the same time point. Scale bar, 100 *μ*m.

The CNV lesions were stained with HE 14 and 21 days after laser photocoagulation, as shown in Figure 6. In the photocoagulation lesions of the vehicle-treated group, depigmentation was observed in the RPE, and CNV had formed in the retinal neuroepithelial layer (RNL), which disrupted Bruch's membrane. Macrophage aggregation and neovascularization between the retinal layers were also observed (Figures 6(a) and 6(c)). The edema, depigmentation, and CNV areas in the CM082-treated group (Figures 6(b) and 6(d)) were significantly decreased compared to those in the vehicle-treated group. The results indicated that CM082 can reduce neovascularization and arrest CNV formation.

**Figure 7:**
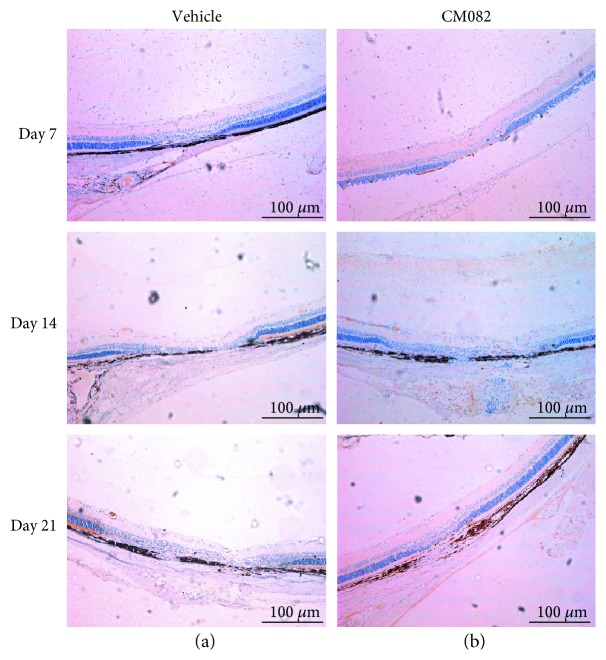
Immunohistochemical analysis of the CM082 (30 mg/kg) group and the corresponding dose of the vehicle group. (a, *n* = 3) The result of immunohistochemical stain with p-VEGFR-2 in the vehicle-treated group at 7, 14 and 21, days after laser photocoagulated. There are large numbers of yellow granules distributed in vessels, outer plexiform layer, and RPE layer. (b, *n* = 3) The expression of p-VEGFR-2 in each structure in the CM082-treated group was significantly lower than the control group at the same time point. Scale bar, 100 *μ*m.

Figure 7 shows that 14 and 21 days after laser injury, phosphorylated VEGFR-2 (p-VEGFR-2) was distributed in the vessels, outer plexiform layer (OPL), and RPE layer in the vehicle-treated group (Figures 7(a) and 7(c)), whereas CM082 administration successfully reduced the aggregation of p-VEGFR-2 in the RPE and OPL (Figures 7(b) and 7(d)). These results suggested that suppressing VEGFR-2 phosphorylation is one of the mechanisms by which CM082 inhibits CNV.

#### 3.2.3. Results of the RPE-Choroid-Sclera Preparations

**Figure 8:**
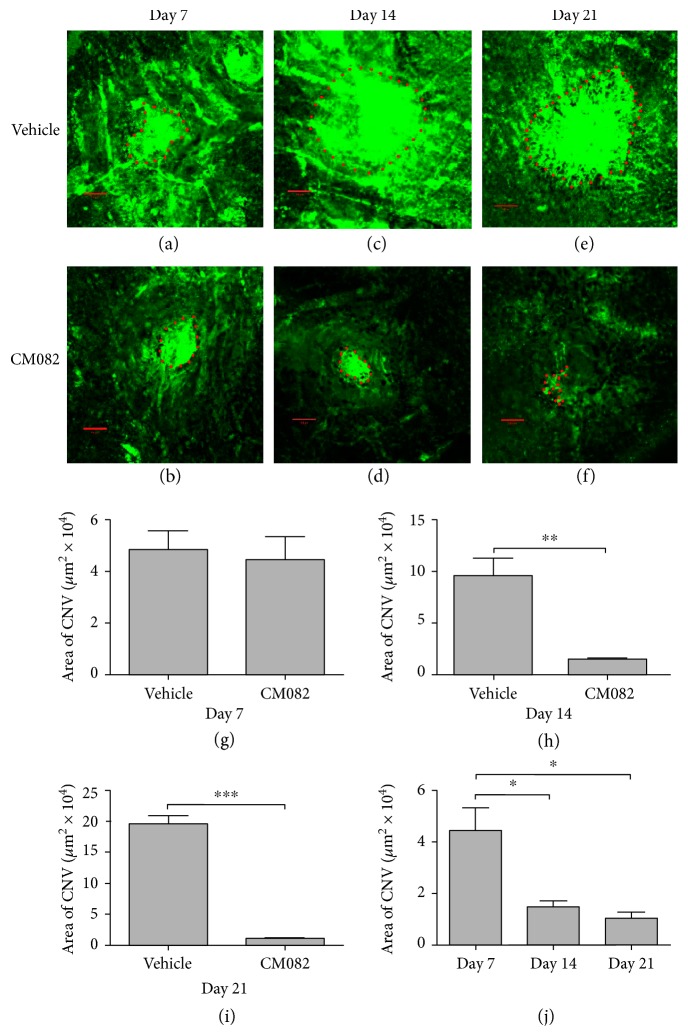
Representative CNV lesions in RPE-choroid-sclera flat mounts by perfusion of fluorescein isothiocyanate-dextrcan were obtained 7, 14, and 21 days after photocoagulation. (a, b) The areas of CNV lesions in the vehicle-treated and CM082 group at day 7 were shown and without statistical difference. CM082 (or vehicle) was delivered orally from day 7 (*n* = 3). The area of CNV lesions was significantly reduced under CM082 treatment at day 14 (c, d; *n* = 3) and 21 (e, f; *n* = 3). (h, i) There is a significantly inhibition of CNV area in CM082 group compared with that in vehicle-treated group (^∗∗^*p* < 0.01, ^∗∗∗^*p* < 0.001). (j) The CNV area in the CM082 group showed a regression at day 14 and 21 compared with that in day 7 (^∗^*p* < 0.05). Scale bar, 100 *μ*m.

To further confirm the reverse effect of CM082 at a dose of 30 mg/kg/d on the CNV area, we prepared RPE-choroid-sclera by perfusion with FITC-dextran. For this purpose, another 20 rats were randomly divided into two groups. Seven days after laser photocoagulation, we examined the neovascularization area in each group to confirm that the groups exhibited similar levels. In particular, the areas of the CNV lesions in the vehicle-treated group were similar to those in the CM082-treated group (vehicle: 4.84 ± 0.72 *μ*m^2^ × 10^4^, CM082: 4.45 ± 0.90 *μ*m^2^ × 10^4^; Figures 8(a), 8(b), and 8(g)). The vehicle or CM082 was then administered to each group until 14 or 21 days after laser injury. We demonstrated that the areas of the CNV lesions in the CM082-treated group (14 days: 1.48 ± 0.24 *μ*m^2^ × 10^4^, 21 days: 1.03 ± 0.27 *μ*m^2^ × 10^4^) were significantly decreased compared to those in the vehicle-treated group (14 days: 9.60 ± 1.68 *μ*m^2^ × 10^4^, 21 days: 19.61 × 10^4^; Figures 8(c) – 8(i); ^∗∗^*p* < 0.01, ^∗∗∗^*p* < 0.001). In addition to comparing the CM082-treated group to the vehicle-treated group, we analyzed the CNV area in the CM082-treated group at different time points. The results indicated that the CNV lesions had significantly regressed following CM082 administration (Figure 8(j); ^∗^*p* < 0.05). All these results confirmed the FFA results and demonstrated the regression of CNV lesions following CM082 treatment.

## 4. Discussion

In our study, we investigated the effect of a novel receptor tyrosine kinase inhibitor (CM082) on CNV and determined the concentration of CM082 in the eyes of BN rats. The results for both the extent and the areas of the CNV lesions in the CM082-treated group were significantly decreased and demonstrated regression compared to the results observed for the vehicle-treated group. The reduced expression of p-VEGFR-2 in the CM082-treated group compared to the vehicle-treated group indicated that VEGF signaling was inhibited by CM082 in the retina-choroid tissues of the CNV rat model. As previously reported, the most common function of VEGF is promotion of neovascularization, more so than maintaining existing vessels. Thus, we found a regressive effect of CM082 on early established CNV that may not be due to VEGF blockade. Because CM082 plays a role in inhibiting PDGF signaling, we conjecture that CNV regression is a result of PDGFR inhibition and pericyte dysfunction. However, Figure 1(e) showed that CM082 treatment started from day 22 exerted little efficiency. It indicated that CM082 should be treated at an early stage of CNV formation. Subsequent research will be needed to determine the importance of the mechanism of PDGF signaling inhibition by CM082 in neovascular regression and normal vessel stabilization. The pharmacokinetics and distribution results showed that there was no significant difference in the CM082 concentrations in the OS, OD, and plasma. It demonstrates that CM082 was absorbed rapidly and passed the BRB to affect the retina following oral administration. The high concentrations of CM082 in the retina and choroid could be therapeutically beneficial for exudative AMD, whereas the low concentration in the aqueous fluid may be explained by the low solubility of CM082 in tears and other liquid contained in the aqueous fluid. The results of our study demonstrated that CM082 can inhibit CNV formation and effectively induce regression of established CNV following oral administration. After oral administration, CM082 is equally distributed to the eyes and is efficiently absorbed. This is the first report of the effects of this treatment in a CNV animal model.

As previously mentioned, the pathology of CNV is not completely understood and the therapeutic methods are limiting. Certain recent clinical trials have reported that intravitreal injections of a VEGF inhibitor can arrest type 1 CNV progression, which reduces the central thickness of the retina and efficiently prevents vision loss in patients with wet AMD [24, 25]. Currently, anti-VEGF therapies, such as ranibizumab and bevacizumab, have become the main treatment for exudative AMD in the clinic. However, these therapies cannot induce regression of established, type 2 CNV, and repeated injections of anti-VEGF treatments may cause a number of complications, including vision impairment, media opacification, and intraocular inflammation [26]. Moreover, some individuals do not respond to intravitreal anti-VEGF drugs, so their vision is not improved over baseline [27]. The underlying mechanism may be due to the complex interaction of VEGFs and VEGFRs. Ranibizumab and bevacizumab are antibodies that target VEGF-A. However, VEGF-D and VEGF-E also play roles in neovascularization by binding VEGF receptors [28, 29]. Besides, studies have noticed PDGF, another critical factor in angiogenesis. It is a crucial molecule in vessel progression and stabilization that binds to PDGFR [19]. Previous studies indicated that treatments that simultaneously inhibit VEGFRs and PDGFRs may not only suppress neovascularization but also cause the regression of established vessels [20, 30]. Consequently, simultaneous inhibition of VEGF and PDGF may contribute to reducing nonresponsiveness.

CM082 is an orally bioavailable small-molecule inhibitor of all isoforms of VEGFRs and PDGFRs, with antiangiogenic and antineoplastic effects. Its design was based on sunitinib. Preclinical studies have indicated that sunitinib can inhibit corneal neovascularization [31]. Vatalanib, pazopanib, and sorafenib are tyrosine kinase inhibitors similar to sunitinib. Vatalanib can inhibit PDGFR and c-kit and has been confirmed to inhibit neovascularization [32]. Pazopanib can inhibit VEGFR, PDGFR, and c-kit. A phase 1/2 clinical trial has demonstrated that this drug exhibits promising antitumor activity and has a favorable toxicity profile [33]. This therapy has also been tried in the form of eye drops or as an oral medicine to treat exudative AMD [34, 35]. These studies indicate that this type of multitarget receptor tyrosine kinase inhibitor can significantly suppress CNV development. Tyrogenex has completed a phase 1 clinical trial of CM082 and is conducting a phase 2b study in patients with exudative AMD. In a model of oxidative-induced retinopathy, the inhibition rate in a CM082-treated group reached 71.1% compared to that in a control group. Results from another CNV model produced by subretinal injection of Matrigel also indicated that CM082 reduced the areas of CNV lesions.

In conclusion, oral administration of 10 mg/kg/d or 30 mg/kg/d CM082 reduces the area of the CNV lesions and pathological neovascularization in a laser-induced CNV model in BN rats. CM082 treatment could reduce the necessity of intravitreal injections and decrease side effects, as oral administration and long-term application are permitted. More thorough pharmacological and therapeutic analyses of CM082 will be required to illustrate the mechanism of PDGF signaling inhibition and the safety and cost.

## 5. Conclusion

CM082 can reach the retina successfully by oral treatment in BN rats. Oral administration of 10 mg/kg/d or 30 mg/kg/d CM082 reduces the area of the CNV lesions and pathological neovascularization in a laser-induced CNV model in BN rats.
